# Novel strategies for inhibiting SufA protease: the role of ester substituents and biological properties

**DOI:** 10.1080/17568919.2025.2545173

**Published:** 2025-08-20

**Authors:** Ewa Burchacka, Paweł Pięta, Katarzyna Pstrowska, Agnieszka Korzenowska-Kowal, Gabriela Cieniuch, Michał Jewgiński

**Affiliations:** a Department of Organic and Medicinal Chemistry, Faculty of Chemistry, Wrocław University of Science and Technology, Wrocław, Poland; b Department of Cell Biology, Poznan University of Medical Sciences, Poznań, Poland; c Department of Advanced Material Technologies, Faculty of Chemistry, Wrocław University of Science and Technology, Wrocław, Poland; d Collection of Microorganisms, Department of Immunology of Infectious Diseases, Hirszfeld Institute of Immunology and Experimental Therapy, Polish Academy of Sciences, Wrocław, Poland; e Department of Bioorganic Chemistry, Faculty of Chemistry, University of Science and Technology, Wrocław, Poland

**Keywords:** Serine proteases, inhibitors, antibacterial treatment, SufA protease, phosphonate

## Abstract

**Aims:**

This study aims to develop novel antibacterial agents by targeting SufA protease, a key virulence factor in Finegoldia magna, using 1-aminoalkylphosphonate (1-AAP) diaryl esters as inhibitors.

**Materials & methods:**

Structural optimization of a reference inhibitor, Cbz-6-AmNpthP(OC₆H₅)₂, was performed by introducing substituents at the para position of phenyl rings: -SCH₃ (**8a**), -OCH₃ (**8b**), and -COOCH₃ (**8c**). Enzymatic assays, molecular modeling, antibacterial activity screening, and CD spectroscopy were utilized to evaluate inhibitory potency, binding interactions, functional effects, and DNA interaction.

**Results:**

Compound **8a** demonstrated moderate SufA inhibition (k₂/K_i_ = 1500 M^− 1^ s^− 1^), supported by molecular modeling that showed stable binding via hydrogen bonding and π–π stacking. It also protected host defense molecules (fibrinogen, LL-37) and exhibited broad-spectrum antibacterial activity (IC₅₀ = 0.02 µM against S. marcescens and F. magna). Compound 8c, despite weak SufA inhibition, displayed potent antibacterial activity (IC₅₀ < 0.01 µM), surpassing gentamicin.

**Conclusions:**

1-AAP derivatives, particularly 8a and 8c, exhibit promising antibacterial properties. These findings validate SufA as a therapeutic target and support further development of peptide-based inhibitors to enhance efficacy and selectivity.

## Graphical abstract

**Figure:**
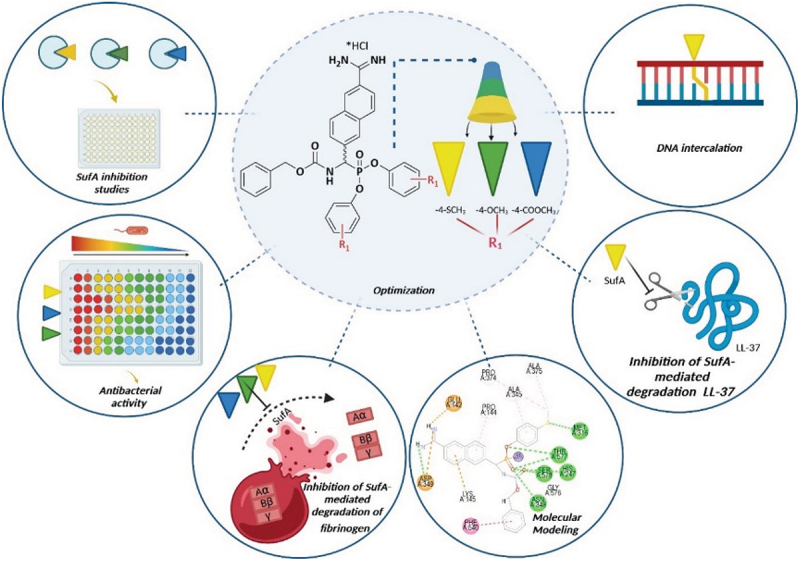

## 1. Introduction

*Finegoldia magna* is considered the most pathogenic species among gram-positive anaerobic cocci (GPAC) [1]. GPAC typically constitute about 25–30% of anaerobic bacteria isolated from clinical samples, while *F. magna* is often found in polymicrobial infections and is also the most frequently isolated GPAC in pure culture from various clinical samples [2]. *F. magna* is one of the most commonly isolated anaerobes from the skin in infections such as wound infections, soft tissue abscesses, diabetic and pressure ulcers, endocarditis, pneumonia, osteomyelitis, and bone and prosthetic joint infections, as well as in septic arthritis [3–5]. Fatal cases of *F. magna* infections have been observed in monomicrobial bacteremia and native valve endocarditis leading to left ventricular rupture [6]. Furthermore, this species has been associated with cases of necrotizing fasciitis, necrotizing pneumonia, mediastinitis, toxic shock syndrome, and meningitis [6]. The occurrence of multidrug-resistant strains is on the increase despite the susceptibility of *F. magna* strains to many antibiotics belonging to β-lactams, tetracyclines, quinolones, or macrolides. One of the virulence factors of *F. magna* is the cell wall-associated protease SufA, a key extracellular subtilisin-like serine proteinase. SufA is a subtilisin-like protease that features a catalytic triad consisting of Asp, His, and Ser. The extracellular activity of SufA protease contributes to: (1) increased penetration into deeper tissues, and the spread of infection by degrading extracellular matrix components; (2) evasion of immune mechanisms by degrading antimicrobial peptides and modulating chemokine activity, (3) delayed wound healing caused by inhibiting fibrin network formation, (4) inactivation of innate immune system factors by interacting with other virulence factors such as FAF and protein L [7–9].

Derivatives of 1-aminoalkylphosphonate (1-AAP) diaryle esters are low molecular chemical compounds that inhibit the enzymatic activity of serine proteases. The mechanism of 1-AAP inhibition of serine proteases has been well understood and occurs via an attack of the hydroxyl group of serine on the phosphorus atom [10,11]. The literature data indicate that changes made to the structure of diphenyl esters of 1-AAP by introducing substituents to phenyl rings affect the inhibitory activity toward proteases [12,13]. Introducing -S-CH_3_ on the phenyl rings in *para* position to tripeptide derivatives Boc-Val-Pro-Val^P^(OC_6_H_5_)_2_ increases its inhibitory activity toward human neutrophil elastase by over four times [13]. In the case of phenylalanine-derived phosphonate analogs targeting subtilisin, compounds with a -COOCH₃ substituent demonstrated a k₂/Ki value of 730 M^− 1^ s^− 1^, while the -SCH₃ derivative showed 802 M^− 1^ s^− 1^, and the unsubstituted compound yielded 760 M^− 1^ s^− 1^ [12]. These results suggest that such electron-donating or electron-withdrawing groups may subtly modulate inhibitory activity. More striking effects were observed for phenylalanine phosphonate analogs targeting chymotrypsin. Among the most potent inhibitors selected for SAR analysis were those with -OCH₃ (k₂/Ki = 76000 M^− 1^ s^− 1^), -COOCH₃ (k₂/Ki = 300000 M^− 1^ s^− 1^), and -SCH₃ (k₂/Ki = 86000 M^− 1^ s^− 1^) substituents [12]. In contrast, the unsubstituted analog exhibited significantly lower activity (k₂/Ki = 3460 M^− 1^ s^− 1^) [12]. These data underscore the relevance of these substituents in enhancing the inhibitory potency of phosphonate-based compounds and provide a rationale for their inclusion in our study. Accordingly, the selection of -SCH₃, -OCH₃, and -COOCH₃ substituents in our inhibitors was informed by their proven efficacy in related systems, aiming to explore similar effects in the context of SufA protease inhibition.

The investigation of the inhibitory activity of chemical compounds toward specific proteins is a popular approach in research on inhibitors [13–15]. The selection of the most active inhibitor usually leads to the extensive modifications of compound structure to improve the specificity and selectivity of its action. In our recent study, Cbz-6-AmNpth^P^(OC_6_H_5_)_2_, an amidine naphthalene derivative of diphenyl esters of 1-AAP, demonstrated inhibitory activity against SufA, with a k_2_/K_i_ value of 10,800 M^− 1^ s^− 1^ and completely inhibited the degradation of fibrinogen [16]. Moreover, Cbz-6-AmNpth^P^(OC_6_H_5_)_2_ exhibited antibacterial activity (MIC range from 1.4 to 14 mg/L), while showing no significant cytotoxicity toward cell lines of BALB/3T3 and HLMEC [16]. However, the amidine substituent is also common to several well-known DNA-binding molecules [17–23]. In this work, we assumed the possibility of undesirable interactions of phosphonate inhibitor derivative with DNA. We also presented the design and synthesis of inhibitors of SufA protease with antibacterial activity. The structure of the SufA protease inhibitor Cbz-6-AmNpth^P^(OC_6_H_5_)_2_ was optimized by introducing functional groups in the position of the aryl group, and the effect of new functional groups on inhibiting the catalysis of the enzymatic reaction was investigated. We also demonstrated profiling for inhibitors toward inhibition of SufA-mediated degradation of human LL-37 and fibrinogen.

## 2. Experimental section

All reagents used in the experiments were purchased from commercial suppliers. The SufA protease and ABZ-Ile-Ser-Lys-ANB substrate for enzymatic studies originated from Lund University (Sweden) and the University of Gdańsk (Poland), respectively. The DNA was obtained from the thymus of an ox from the Hirszfeld Institute of Immunology and Experimental Therapy, Polish Academy of Sciences (Wrocław, Poland). ^1^ H and ^3 1^ P nuclear magnetic resonance (NMR) spectra were recorded on either a 400 MHz Jeol PCZ 400S NMR spectrometer (Jeol, Warsaw, Poland) or a 600 MHz Bruker Avance spectrometer (Bruker). Enzyme assays were performed with a SpectraMax Gemini XPS spectrofluorometer (Molecular Devices, Sunnyvale, CA, USA). High-resolution mass spectra were recorded with a Waters Acquity Ultra Performance LC, LCT Premier/XE system (Waters, Warsaw, Poland) or an HPLC-MS Thermo Scientific Ultimate 3000 instrument with a vacuum degasser (Thermo Scientific Inc., USA). HPLC analysis was performed by means of an Interchim® C18 HQ-250/212 reverse-phase column (250 × 4.6 mm, 5 μm) and HPLC-grade solvents: acetonitrile and water with 0.1% formic acid. The sample injection volume was 10 μL, the flow rate was 1 mL/min, and the detection wavelengths were 280 and 254 nm. Preparative high-resolution liquid chromatography was done using a Waters Binary Module System (Waters) with a dual λ absorbance detector system, employing an Interchim® C18 HQ-250/P46 reverse-phase column (250 mm × 21.2 mm, 10 μm) with a flow rate of 15 mL/min and a linear gradient from 0 to 100% B within 20 min (solvent A: H₂O with 0.05% trifluoroacetic acid, solvent B: MeCN with 0.05% trifluoroacetic acid). Circular dichroism (CD) spectra of DNA and DNA – inhibitor complexes were measured with a Jasco J-1500 CD spectrometer. The antibacterial activity of inhibitors against *F. magna* PCM 2822 was determined at the Polish Collection of Microorganisms, Hirszfeld Institute of Immunology and Experimental Therapy, Polish Academy of Sciences. Growth of *F. magna* PCM 2822 in the presence of inhibitors was monitored using a Tecan Spark® Multimode Microplate Reader. Antibacterial activity of inhibitors 8a-c against *Staphylococcus aureus* PCM 2602, *Serratia marcescens* PCM 549, and *Escherichia coli* PCM 2561 was evaluated at Wrocław University of Science and Technology, and absorbance was recorded by means of a Thermo® Multiscan FC Photometer 357.

### 2.1. Inhibitor synthesis

Detailed descriptions of the synthetic procedures and spectroscopic data for intermediate compounds are provided in the Supplementary Material (Supplementary Chemical Part S1-S2 and Figures S1–S8). Mass spectrometry and nuclear magnetic resonance analyses of final inhibitors 8a-c are described below. The synthesis of all tested inhibitors began with the preparation of 6-formyl-2-naphthonitrile (Figure S8, stage 1). Briefly, dimethyl 2,6-naphthalenedicarboxylate (1) was first selectively hydrolyzed to 6-(methoxycarbonyl)-2-naphthoic acid (2). Next, the carboxylic group was transferred into an amide, and subsequent dehydration led to nitrile formation. The ester group was then reduced to an alcohol and oxidized under Swern conditions to the corresponding aldehyde (6). The resulting 6-formyl-2-naphthonitrile (6) was used for the amidoalkylation reaction of appropriately substituted triphenyl phosphite with benzyl carbamate, leading to Cbz-(6-CN)Nphth^P^(OC_6_H_4_-R1)_2_ (7, Figure S8, stage 2) [24,25]. In the next step, the imino ether was produced under anhydrous conditions and then transformed into an amidine group using ammonium in methanol.

Cbz-6-AmNphth^P^(OC_6_H_4_-4-SCH_3_)_2_ (**8a**): ^31^P NMR (243 MHz, Methanol-d4) δ 14.14; ^1^H NMR (601 MHz, Methanol-d4) δ 8.42–6.81 (m, 7H), 5.77 (d, J = 23.1 Hz, 1H), 5.12 (d, J = 12.4 Hz, 1H), 4.98 (d, J = 12.3 Hz, 1H), 2.31 (d, J = 5.7 Hz, 6H); HPLC-MS m/z calcd. for C_34_H_32_N_3_O_5_PS_2_ (M+H)+ 658.2, found 658.4

Cbz-6-AmNphth^P^(OC_6_H_4_-4-OCH_3_)_2_ (**8b**): ^31^P NMR (243 MHz, Methanol-d4) δ 14.35; ^1^H NMR (601 MHz, Methanol-d4) δ 8.38–6.61 (m, 19H), 5.73 (d, J = 23.2 Hz, 1H), 5.13 (d, J = 12.4 Hz, 1H), 4.99 (d, J = 12.3 Hz, 1H), 3.62 (d, J = 3.5 Hz, 6H); HPLC-MS m/z calcd. for C_34_H_32_N_3_O_7_P (M+H)+ 626.2, found 626.4

Cbz-6-AmNphth^P^(OC_6_H_4_-4-COOCH_3_)_2_ (**8c**): ^31^P NMR (243 MHz, Methanol-d4) δ 13.79; ^1^H NMR (601 MHz, Methanol-d4) δ 8.37–6.95 (m, 19H), 5.89 (d, J = 22.9 Hz, 1H), 5.10 (d, J = 12.4 Hz, 1H), 4.96 (d, J = 12.4 Hz, 1H), 3.77 (d, J = 8.4 Hz, 6H); HPLC-MS m/z calcd. for C_36_H_32_N_3_O_9_P (M+H)+ 682.2, found 682.3

### 2.2. SufA inhibition studies

The assays were performed at 37°C with an excitation wavelength of 320 nm for ABZ and an emission wavelength of 410 nm using 96-well microtiter plates (COSTAR, Corning Inc., Gdańsk, Poland) in 0.1 M Tris-HCl (pH 8.0) with 0.01 M KCl. The concentrations of substrate (ABZ-Ile-Ser-Lys-ANB) and SufA protease were 25 µM and 4 µg/mL, respectively. The SufA protease was incubated for 10 min with the tested compound, dissolved in DMSO (inhibitor final concentration ranging from 0 to 300 µM), and then the measurement was taken for 10 min. Each assay was performed in at least duplicate. The inhibitory activity was measured, and the percentage of inhibition was calculated with the equation:

$$\%\;\;\mathrm{inhibition}\;=\;\left[\mathrm{RFUtest}\;/\;\mathrm{RFU}\left(-\right)\right]\;*\;100\%,$$

where RFU represents the relative fluorescence units, test refers to the signal from reactions containing an inhibitor, and (-) represents the signal from the control reactions (without an inhibitor). The inhibitory effects of the synthesized compounds were evaluated by means of the progress curve method. Time-dependent inhibition of HNE resulted in nonlinear progress curves, which were analyzed using the following equation to determine the observed first-order rate constant (k_obs_):

$$\left[P\right]=v_{\mathrm{st}}+\frac{v_{i}-v_{\mathrm{st}}}{k\mathrm{obs}}\left[1-e^{\left(-k_{\mathrm{obs}}\cdot t\right)}\right],$$

where [P] is the product concentration at time t, v_i_ is the initial velocity, and v_s_ is the steady state velocity. To estimate the second-order rate constants (k_2_/K_i_), the values of k_obs_ were divided by the inhibitor concentration ([I]), and the results were corrected for substrate concentration ([S]) and the Michaelis – Menten constant (KM) described by the following equation:

$$\frac{k_{\mathrm{obs}}}{\left[I\right]}=\frac{k_{2}}{K_{i}\left(1+\frac{\left[S\right]}{\mathrm{KM}}\right)}$$

### 2.3. DNA intercalation procedure

The measurement parameters were set as follows: the wavelength range was set from 220 nm to 320 nm, step – at every 0.2 nm, and 4 accumulations. Measurements were performed in a Justcode M cuvette with an optical path of 1 mm. 200 μL of the analyzed solution was introduced into the cuvette each time. The apparatus was zeroed using phosphate buffer with the following composition: 150 mM NaCl, 20 mM PB, 1 mM EDTA. Afterward, the samples (maintaining a constant DMSO concentration of 1%) of ctDNA (50 µM), inhibitor (100 µM), and inhibitor (100 µM) with ctDNA (50 µM) were prepared. First, a sample of the ctDNA solution was measured with a CD spectrometer to check the graph for pure DNA. Then, a sample of the inhibitor solution was measured, against which the apparatus was blanked because the inhibitor molecule itself could produce a CD effect, potentially falsifying the results. At the end, a sample of the actual solution containing the inhibitor and ctDNA was measured.

### 2.4. Inhibition of SufA-mediated degradation of human antimicrobial LL-37 and fibrinogen

The SufA protease was incubated with the specific SufA inhibitor for 60 min at 37°C, maintaining a constant DMSO concentration of 1%. The reference sample contained an appropriate amount of DMSO instead of the inhibitor. After incubation, fibrinogen and LL-37 proteins were added to each sample of enzyme (25 µg/mL) or enzyme with inhibitor (25 µg/mL, 1.3 mM). Samples were analyzed by PAGE using the standard Laemmli protocol for fibrinogen and the procedure developed by Judd for the LL-37 peptide. Raw gel results of the inhibition of SufA-induced degradation of human fibrinogen and LL-37 by tested inhibitors after SDS-PAGE electrophoresis analysis are presented in Supplementary Biochemical Part S1-S2 and Figures S9–S11.

### 2.5. Antibacterial activity

The antibacterial properties of the inhibitors were assessed in Mueller-Hinton (MH) broth at 37°C for *E. coli*, *S. marcescens*, and *S. aureus*. For *F. magna*, the antibacterial properties of the inhibitors were tested in Todd Hewitt broth at 37°C under anaerobic conditions. An overnight culture of the tested bacteria was diluted 200-fold in fresh medium and incubated at 37°C until the OD600 reached 0.3. Subsequently, 100 µL of the inhibitor and antibiotic control (at concentrations ranging from 0 to 250 µg/mL) were added to 900 µL of bacterial culture, which had been previously diluted to an OD600 of less than 0.01. The resulting samples were incubated for 24 h at 37°C with shaking at 100 rpm. Then, 200 µL of each sample was transferred to a well plate, and the absorbance was measured at a wavelength of 600 nm using a microplate reader. To determine the antibacterial properties of the inhibitors, the data were normalized against a control culture (without inhibitor or antibiotics). The IC_50_ values were calculated using GraphPad Prism through nonlinear regression, employing the following equation model:

$$Y=\mathrm{YBottom}+(\mathrm{YTop}-\mathrm{YBottom})/(1+10^{\wedge }((\mathrm{Log}\;IC_{50}-X)*(-1.0))),$$

where YBottom and YTop represent the plateaued units of the Y-axis. The IC_50_ value represents the concentration that produces a half-maximal response between YBottom and YTop, thus measuring the potency of a compound in inhibiting bacterial viability and indicating a 50% reduction in bacterial growth. The effect of gentamicin on bacterial growth reduction, including its IC_50_ values, can be found in the Supplementary Material (Supplementary Microbiology Part S1, Figures S12)

### 2.6. Molecular modeling

The protein structure with UniProt accession code A6XFB7 was utilized in docking. Protein structure was prepared using the Protein Preparation module with simultaneous optimization of hydrogen atom positions. The studied inhibitors 8a-c were optimized with the OPLS-2005 force field [26]. The optimization protocol operated on the LBFGS method with a maximum of 2500 iterations and water as the solvent. For each inhibitor, both R and S isomers were considered. Covalent docking was simulated with the CovDock module using the addition of the predefined reaction type Phosphonate to Ser578 residue [27]. Detailed information describing the formation of phosphonate ester from condensation of ligand phosphonate with receptor alcohol is shown in SI. For each inhibitor, the formation of a phosphonic bond was considered using each of the hydroxyl groups (R1 and R2 and S1 and S2). For this purpose, the appropriate monoester forms of each inhibitor were applied for docking. For each docking with the PosePrediction mode, additional optimization of the resultant complexes was performed within a radius of 3Ang from the docked inhibitor. In addition, the position of ligands was assessed with the MMGBSA score. Protein-inhibitor interactions were analyzed using both the Ligand Interaction module of the Schrodinger package and the Discovery Studio Visualizer program.

## 3. Result and discussion

### 3.1. SufA inhibition studies

**Figure 1.**
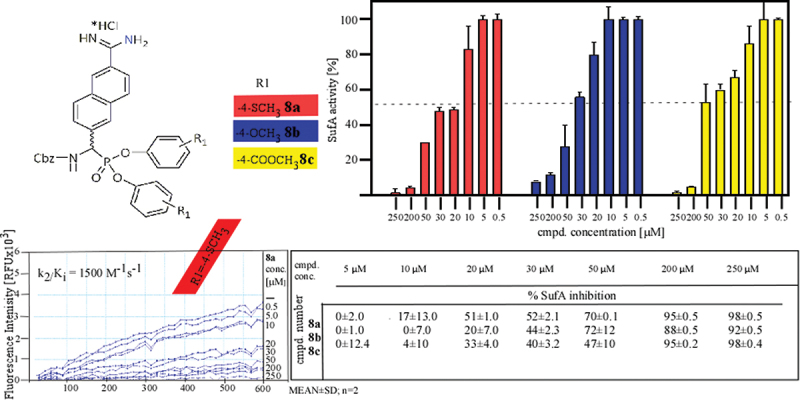
The influence of substituents R1: −4-SCH_3_, −4-OCH_3_, and −4-COOCH_3_ of Cbz-6-AmNpth^P^(OC_6_H_4_-R1)_2_ on the percent of inhibitory activity against SufA protease carried out at different concentrations of inhibitors.

The optimization of the structure of the protease SufA inhibitor Cbz-6-AmNpth^P^(OC_6_H_4_-R1)_2_ was performed by introducing functional groups at the R1 position: −4-SCH_3_ (**8a**), −4-OCH_3_ (**8b**), and −4-COOCH_3_ (**8c**). All synthesized analogs were obtained as racemic mixtures and inhibited SufA protease, with compound 8a, a 4-mercaptomethylphenyl ester derivative, exhibiting the inhibitory potency, achieving k_2_/K_i_ value of 1500 M^−1^s^−1^ (Figure 1). Compound 8a, bearing a 4-mercaptomethyl (-SCH₃) substituent on the phenyl ring, emerged as the most potent inhibitor of SufA protease among the synthesized analogs. Despite exhibiting approximately ten-fold lower inhibitory activity than the unsubstituted reference compound (k₂/K_i_ = 10800 M^− 1^ s^− 1^), 8a still displayed significant inhibitory potential, with a k₂/K_i_ value of 1500 M^− 1^ s^− 1^, indicating a favorable interaction with the SufA active site. Importantly, this result highlights that subtle modifications of the aryl moiety in the diphenyl ester can retain or modulate activity even without strong electron-withdrawing properties. Unlike electron-withdrawing groups that primarily influence reactivity via electronic effects, the -SCH₃ group may exert its influence through favorable hydrophobic interactions or potential van der Waals contacts within the S1′ or S2′ binding pockets of the enzyme. Compound 8b and 8c showed lower inhibitory activity than 8a, therefore it was not possible to determine the kinetic parameters k_2_/K_i_ by their crystallization at higher concentrations. Surprisingly, compound 8c displayed the lowest inhibitory potency toward SufA protease among the tested inhibitors. Despite the strong electron-withdrawing properties of the 4-carboxymethyl group, compound 8c did not inhibit even 50% of the protein activity at 50 µM (Figure 1). Literature data indicate that for most serine proteases, diphenyl esters of 1-AAP with electron-withdrawing substituents enhance enzyme inhibition potency [12,28,29]. For instance, phosphonate tripeptides Cbz-Val-Pro-Leu^P^(OC_6_H_4_-4-SOOCH_3_)_2_ and Cbz-Val-Pro-Phe^P^(OC_6_H_4_-4-SOOCH_3_)_2_ exhibited the highest inhibitory potency toward SplA protease, with a k_2_/Ki value > 8000 M^−1^s^−1^ [28]. The effect of substituents was also evaluated against subtilisin and chymotrypsin, where the introduction of the −4-SOOCH_3_ substituent into the diphenyl ester of Cbz-Phe^P^(OC_6_H_4_-R1)_2_ resulted in high inhibitory activity against both enzymes, with k_2_/Ki values of 47,100 ± 2305 M^−1^s^−1^ and 317,380 ± 21500 M^−1^s^−1^, respectively [12]. Similarly, the introduction of −4-COOCH_3_ and −4-SOOCH_3_ substituents significantly improved the human neutrophil elastase inhibitory activity of the Cbz-Val^P^(OC_6_H_4_-R1)_2_ derivative, with k_2_/Ki values of 33,015 ± 1445 M^−1^s^−1^ and 15,945 ± 285 M^−1^s^−1^, respectively [29].

However, the practical application of these compounds with electron-withdrawing substituents is limited due to their increased susceptibility to hydrolysis during the initial metabolic steps. Derivatives 8a (4-mercaptomethylphenyl ester) and 8b (4-methoxyphenyl ester) lack an electron-withdrawing moiety, and their activity most likely results from additional interactions within the enzyme’s leaving group binding site (S1‘, S2’ pockets). Nonetheless, their inhibitory potency toward SufA protease was not significantly superior to the unsubstituted ester derivative. It appears that substitution at the phenyl diester ring does not drastically enhance activity against SufA protease. In other words, the leaving group binding site of SufA may be relatively small and does not readily accommodate the phenyl rings of the tested substituents.

### 3.2. DNA intercalation

Chemical compounds capable of DNA intercalation are characterized by a planar structure with a thickness between 2 and 3.7 Å, which significantly facilitates their insertion between base pairs [30]. Examples of DNA intercalators include N-acyloxy-N-alkoxyamides attached to a naphthyl group and various dicationic amidine compounds that bind within the DNA minor groove [31,32]. The structural similarity of these compounds to the synthesized SufA protease inhibitors raised the hypothesis that this group of compounds might, in addition to inhibiting SufA protease, also exhibit antibacterial activity through DNA intercalation. Such an effect in a potential antibacterial drug could, on one hand, enhance bactericidal activity, but on the other, could negatively impact host cells, potentially leading to mutagenesis due to intercalation with human host cell DNA. Structural changes occur in the oligonucleotide molecule upon intercalator insertion into its tertiary structure. Ligand insertion results in DNA molecule elongation, with the distance between adjacent base pairs in the intercalated complex increasing from 3.4 Å to 7 Å [33]. Partial double helix unwinding also occurs, with the twist angle decreasing from 10° to 26° [30]. The effect of compound 8a, selected for its high SufA protease inhibition activity on DNA structure changes, was assessed using circular dichroism (CD) spectrometry. Circular dichroism (CD) spectroscopy was selected as the method for investigating potential DNA intercalation due to its high sensitivity to conformational changes in nucleic acids upon ligand binding. This technique enables rapid and noninvasive analysis of alterations in the secondary structure of DNA in solution, without the need for sample labeling or complex preparation procedures [34]. Compared to other methods such as UV-Vis spectroscopy, fluorescence spectroscopy, or calorimetry, CD provides direct insights into changes in DNA helicity and base stacking interactions – critical parameters for identifying intercalative binding modes [34]. The ability of CD to detect subtle modifications in the chiral environment of DNA makes it particularly suitable for evaluating whether the tested compounds interact with DNA in a biologically relevant manner. For these reasons, CD spectroscopy was considered the most appropriate technique to assess the nature of compound – DNA interactions in this study.

**Figure 2.**
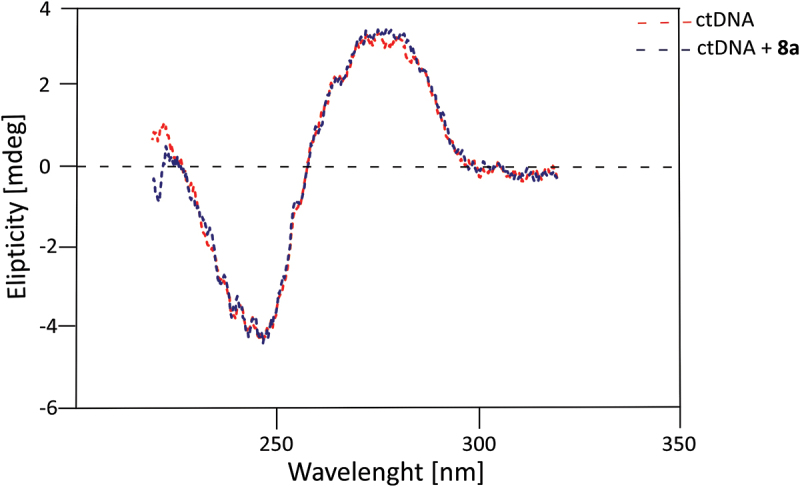
The molar ellipticity circular dichroism spectra of ctDNA (red dashed line) and ctDNA with 8a (blue dashed line).

The standard CD spectrum for right-handed duplex circulating tumor DNA (ctDNA) exhibits a maximum at 280 nm, a minimum at 250 nm, and a crossover point at 260 nm [30]. The CD spectra of ctDNA and ctDNA with compound 8a are shown as red and blue lines, respectively (Figure 2).

Analysis of the ctDNA spectrum and the spectrum of ctDNA with compound 8a reveals almost complete overlap. The only discrepancy occurs in the 220 nm to 225 nm range, but this difference is not significant, as intercalation would induce substantial changes in the CD effect intensity at the maximum and minimum [30]. Based on these results, it can be concluded that compound 8a does not induce DNA intercalation.

### 3.3. Inhibition of SufA-mediated degradation of human antibacterial LL-37 and fibrinogen

**Figure 3.**
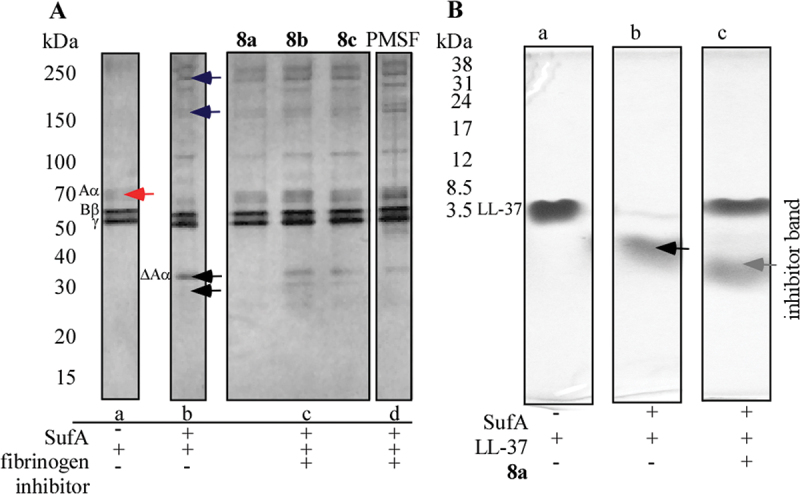
A) inhibition of SufA-induced degradation of human fibrinogen by tested inhibitors a) human fibrinogen b) human fibrinogen incubated with SufA c) human fibrinogen incubated with SufA and inhibitor: 8a (line 1), 8b (line 2), 8c (line 3) d) human fibrinogen incubated with SufA and PMSF. The red arrow indicates the fibrinogen Aα chain, while the black arrows highlight the degradation products of the Aα chain. The blue arrow indicates the SufA protease. B) inhibition of SufA-induced degradation of antibacterial peptide LL-37 by tested 8a a) LL-37 b) LL-37 incubated with SufA c) LL-37 incubated with SufA and 8a. The black arrow highlights the degradation products of the LL-37. The gray arrow highlights the inhibitor band (Supplementary biochemical Part S2, Figures S11).

To assess the ability of compounds 8a-c to inhibit the degradation of human fibrinogen by SufA, purified fibrinogen was subjected to testing. Following pre-incubation of SufA with the inhibitors and subsequent addition of fibrinogen, the samples were analyzed by SDS-PAGE under reducing conditions (Figure 3(A)). SufA exhibited prominent bands at approximately 250 kDa and weaker bands around 130 kDa, as depicted in Figure 3(b–c). Human fibrinogen comprises three pairs of polypeptide chains – Aα, Bβ, and γ—linked by disulfide bonds within a 340 kDa complex [35]. Under reducing conditions, these chains appear as bands at approximately 70 kDa, 60 kDa, and 55 kDa, respectively (Figure 3(Aa)).

To confirm the fibrinogen-cleaving activity of SufA, purified fibrinogen was incubated with SufA, resulting in the production of 32 kDa and 35 kDa degradation fragments (Figure 3(Ab)). The results demonstrated that compound 8a completely prevented fibrinogen degradation in purified fibrinogen (Figure 3(Ac)). In the absence of an inhibitor, control SufA samples showed complete degradation of the Aα-band (∼70 kDa), yielding 32 kDa and 37 kDa fragments (Figure 3(Ab)). Compounds 8b and 8c showed a weaker inhibitory effect on fibrinogen degradation, evidenced by the appearance of 32 kDa and 37 kDa bands corresponding to fibrinogen degradation products (Figure 3(Ac)). The results for compounds 8b and 8c were very similar to those obtained with PMSF, which served as a control inhibitor of serine protease activity (Figure 3(Ad)).

Literature studies have demonstrated that SufA degrades antimicrobial peptides, such as LL-37, thereby promoting bacterial survival during infection [8]. LL-37 exhibited bands at 3.7 kDa (Figure 3(Ba)). Pre-incubation of SufA protease with LL-37 confirmed the degradation of the antimicrobial peptide into shorter fragments (Figure 3(Bb)). Pre-incubation of the enzyme with compound 8a resulted in the inhibition of LL-37 cleavage (Figure 3(Bc)).

### 3.4. Effect of the compounds on growth reduction of gram-positive and gram-negative bacteria

**Figure 4.**
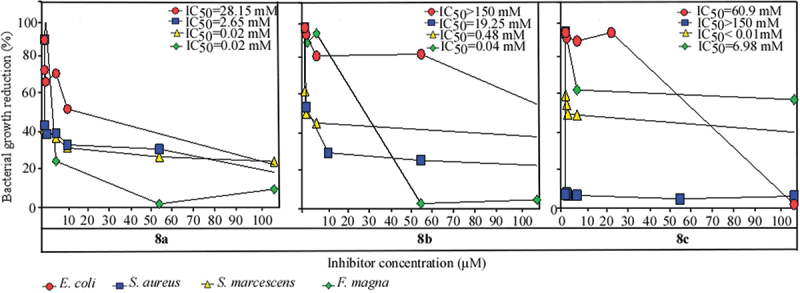
Effect of the compounds 8a-c on the grow reduction of *F. magna* PCM 2822, *S. aureus* PCM 2602, *E. coli* PCM 2561 and *S. marcescens* PCM 549.

Compounds 8a-c were tested for their ability to reduce the growth of gram-positive and gram-negative bacteria: the highly successful opportunistic pathogen *F. magna* PCM 2822, and three strains closely related to those listed among the WHO’s top 16 priority pathogens: *S. aureus* PCM 2602, *E. coli* PCM 2561, and *S. marcescens* PCM 549 [36]. Gentamicin, used as a control in accordance with literature data, inhibited the growth of all tested bacteria (Supplementary Microbiology Part S1). Compound 8a effectively inhibited the growth of both Gram-positive and gram-negative bacteria, with IC_50_ values ranging from 0.02 to 28.15 µM. Notably, the highest antibacterial activity was observed against *S. marcescens* PCM 549 and *F. magna* PCM 2822, with an IC_50_ value of 0.02 µM (Figure 4). Compound 8a also reduced the growth of *S. aureus* PCM 2602 (IC_50_ value of 2.65 µM). Although *S. aureus* PCM 2602 was over 100 times less sensitive than the other tested bacteria, compound 8a still exhibited the highest antibacterial potency against *S. aureus* PCM 2602 compared to inhibitors 8b-c (IC_50_ of 19.25 µM for 8b, and IC_50_ > 150 µM for 8c) (Figure 4). Compound 8a showed approximately a 10-fold weaker inhibition of *E. coli* PCM 2561 growth compared to *S. aureus* PCM 2602 (IC_50_ value of 28.15 µM) (Figure 4). Consistent with previous observations, compound 8a demonstrated the strongest antibacterial effect against *E. coli* PCM 2561 compared to inhibitors 8b-c (IC_50_ > 150 µM for 8b, and IC_50_ of 60.9 µM for 8c) (Figure 4). A significant effect of the −4-COOCH_3_ substituent in compound 8c was observed against *S. marcescens* PCM 549, where the concentration required to reduce the bacterial count by half was below 0.01 µM (Figure 4). This value suggests that compound 8c possesses greater antibacterial activity than the commercially used antibiotic gentamicin (experimental IC_50_ value of 0.2506 µM) (Supplementary Microbiology Part S1, Figure S12). These results collectively indicate that compound 8a inhibited all tested bacteria, compound 8b particularly inhibited *S. aureus* PCM 2602, and compound 8c exhibited the strongest antibacterial effect against Gram-negative bacteria (*E. coli* PCM 2561 and *S. marcescens* PCM 549) compared to gram-positive bacteria (*S. aureus* PCM 2602 and *F. magna* PCM 2822).

To contextualize the antibacterial potential of compounds 8a – c, their IC₅₀ values against *E. coli* and *S. aureus* were compared with those of commonly used antibiotics, including ampicillin and chloramphenicol. The reference values for these antibiotics come from literature sources and used for direct comparison [37,38]. Compound 8a demonstrated moderate activity against *E. coli* (IC₅₀ = 28.15 µM) and strong inhibitory activity against *S. aureus* (IC₅₀ = 2.65 µM). Although it was approximately three times less potent than ampicillin against *E. coli* (literature IC₅₀ = 10.14 µM [37],), 8a showed significantly stronger activity than chloramphenicol (literature IC₅₀ = 373 µM [38],) in this context. Against *S. aureus*, 8a exhibited activity comparable to chloramphenicol (literature IC₅₀ = 1.59 µM [38],), though it remained less potent than ampicillin (literature IC₅₀ = 0.18 µM [37],). In contrast, compounds 8b and 8c showed considerably weaker antibacterial activity. Compound 8b was only modestly active against *S. aureus* (IC₅₀ = 19.25 µM) and inactive against *E. coli* (IC₅₀ > 150 µM). Compound 8c was inactive against *S. aureus* (IC₅₀ > 150 µM) and showed limited activity against *E. coli* (IC₅₀ = 60.9 µM). These results indicate that compound 8a is most promising among the tested analogs, especially considering its superior performance over chloramphenicol against *E. coli* and its notable efficacy against *S. aureus*. While ampicillin remains generally more potent, the observed activities suggest that compound 8a could serve as a viable lead structure for developing novel antibacterial agents targeting gram-positive and gram-negative bacteria.

### 3.5. Molecular modeling

Molecular modeling methods with covalent docking were used to assess the mode of action of the studied inhibitors with the SurfA protein. Because inhibitors were applied in biological studies as a mixture of R and S isomers, both isomers of each inhibitor were also considered in molecular modeling. During the simulation of covalent docking, the formation of a covalent bond between the hydroxyl group of Ser578 and the phosphonic group was considered via both available oxygen atoms. The docking results, together with the values of the scoring functions and the Gibbs energies of the bond formation, are given in Table 1.

**Table 1.** Results of covalent docking of the investigated inhibitors.

Name	MMGBSA dG Bind*	MMGBSA dG Bind* - average of all isomers	MMGBSA dG Bind (NS)**	MMGBSA dG Bind (NS)** - average of all isomers	H-bond	Salt Bridges
8a_R1	−73.35	−81,99	−96.37	−99,93	1	2
8a_R2	−73.73		−84.56		1	0
8a_S1	−92.33		−111.96		7	2
8a_S2	−88.53		−106.82		3	2
8b_R1	−60.11	−76,80	−76.21	−93,46	1	0
8b_R2	−75.81		−91.32		2	2
8b_S1	−89.38		−106.92		7	2
8b_S2	−81.91		−99.40		3	2
8c_R1	−71.21	−74,35	−86.57	−91,46	3	0
8c_R2	−68.05		−79.70		2	0
8c_S1	−84.45		−105.24		4	2
8c_S2	−76.85		−94.32		3	2

*MMGBSA dG Bind = Complex Receptor – Ligand.

**MMGBSA dG Bind(NS) = Complex Receptor (from the optimized complex) – Ligand (from optimized complex).

**Figure 5.**
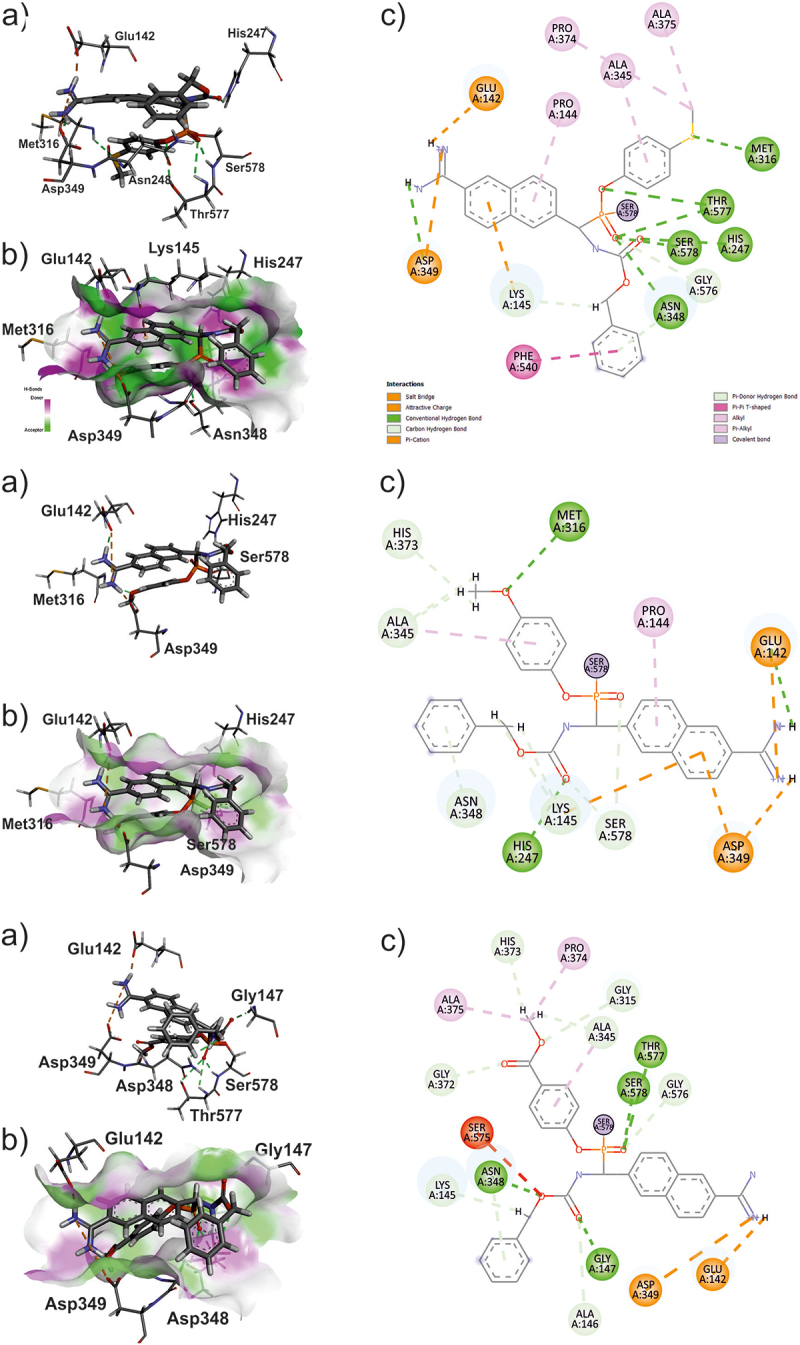
Alignment of 8a (S1) in the active site of SufA a) showed protein residues involved in H-bonds (green dash line)and salt bridges (orange dash line); b) showed protein surface showing acceptor and donor properties; c) showed all inhibitor-protein interaction.

The docking results indicate that the S isomer is preferred regardless of the inhibitor. In the case of each inhibitor 8a-c, a higher binding energy with Ser578 characterizes the S isomers rather than the R isomer. This trend is observable regardless of which oxygen of the phosphonic group is involved in forming the bond (_S1, _S2). One of the factors influencing the interaction between the inhibitors and the protein surface is the orientation of the P(O) group. Only in the case of S-isomers is this group oriented to promote the intermolecular hydrogen bond with the amid proton of Ser578. This additional H-bond further stabilizes the inhibitor alignments in the enzyme’s active site, increasing the binding energy. Since it was impossible to determine the content of individual isomers for each tested compound, we assumed their equimolar amount in the tested samples. Therefore, the average binding energy was calculated for each inhibitor to compare the resultant interaction energy values. The obtained average energies enable us to conclude that the inhibitor that binds most effectively to Ser578 is 8a. As shown in Figure 5 (top panel), the arrangement of 8a_S1 (the best pose found for 8a) in the active cavity of SufA is stabilized by the seven intermolecular hydrogen bonds and two salt-bridge-type bonds. Also, the arrangement of the best pose for the 8b_S1 is stabilized by seven intermolecular hydrogen bonds and two salt-bridge-type bonds (see Figure 5 – middle panel). In contrast, the found pose of the 8c_S1 inhibitor in the SufA active site is stabilized by only four intermolecular hydrogen bonds and two salt-bridge-type bonds (see Figure 5 – middle panel). The lowest number of hydrogen bonds, as well as an unfavorable interaction between the carbonyl oxygen of Ser575 and the oxygen of the Cbz group, results in the lowest MMGBSA energy, which suggests that inhibitor 8c is the worst inhibitor of SufA among all tested compounds. Additionally, the lower affinity toward the enzyme active site could be attributed to the presence of the methyl ester moiety in the *p*-position of the phenyl ester of the phosphine group. This slightly larger derivative, compared to the methylthioether (**8a**) and methylether (**8b**), results in a more exposed hydrophobic Cbz group, which decreases intermolecular binding energy. Interestingly, only in the case of inhibitor 8a is the interaction energy with the protein of both variants of the S isomer, binding via one or the other oxygen of the phosphine group, characterized by the lowest binding energies above 105 kcal/mol (see Table 1). This, assuming that each of the isomers of each inhibitor occurs in equimolar ratios, allows us to conclude that it is the equal affinity of two of the four potential binding modes to Ser578 of SufA that is the reason for the more potent inhibitory properties of 8a compared to the other compounds tested. Detailed information on the individual interactions and the amino acids of the active cavity of SufA involved in these interactions are illustrated in Figure 5.

## 4. Conclusion

Among all the synthesized phosphonic analogs of Cbz-6-AmNpth^P^(OC_6_H_4_-R1)_2_ with functional groups at the R1 position – including −4-SCH_3_ (**8a**), −4-OCH_3_ (**8b**), and −4-COOCH_3_ (**8c**) – compound 8a exhibited the strongest inhibitory activity against SufA protease, showing k_2_/K_i_ value of 1500 M^−1^s^−1^. Further analysis revealed that 8a prevented the degradation of both fibrinogen and the antimicrobial peptide LL-37. Additionally, compound 8a demonstrated the broadest spectrum of antibacterial activity, with IC_50_ values of 0.02, 2.65, 28.15, and 0.02 µM toward *F. magna* PCM 2822, *S. aureus* PCM 2602, *E. coli* PCM 2561, and *S. marcescens* PCM 549, respectively. The greatest activity against *S. marcescens* PCM 549 and *E. coli* PCM 2561 was shown by compound 8c. No compound completely inhibited the growth of *F. magna* PCM 2822 and *S. aureus* PCM 2602. Since there was no significant intercalation of compound 8a with DNA, in vivo studies might be worth doing/considering. Given the involvement of SufA protease in the pathogenesis of bacterial infections, these inhibitors could help clarify its role in vivo.

Future research will focus on the design and synthesis of peptide-based derivatives incorporating the phosphonate warhead to further improve inhibitory potency and enhance biological efficacy. These novel compounds will be structurally based on the amino acid sequence of the most efficient SufA substrate, allowing for increased binding specificity and catalytic efficiency. Additionally, the introduction of selected substituents at the aromatic moiety will be explored to optimize interactions within the enzyme’s active site and fine-tune physicochemical properties for potential in vivo application.

## Supplementary Material

Supplemental Material

## Data Availability

The data used in this study are available at the following DOI: https://doi.org/10.17605/OSF.IO/6D4AH

## Article highlights

- The structure of the SufA inhibitor Cbz-6-AmNpthP(OC₆H₅)₂ was optimized by introducing functional groups at the R1 position: −4-SCH₃ (compound 8a), −4-OCH₃ (compound 8b), and −4-COOCH₃ (compound 8c)
- Compound 8a showed the strongest inhibitory activity against SufA protease among the synthesized analogs, with a k₂/K_i_ value of 1500 M^−1^ s^− 1^
- 8a effectively prevented the degradation of fibrinogen and the antimicrobial peptide LL-37 by SufA
- 8a exhibited a broad spectrum of antibacterial activity against gram-positive and gram-negative bacteria, including *F. magna*, *S. aureus*, *E. coli*, and *S. marcescens* with highest antibacterial activity against *S. marcescens* PCM 549 and *F. magna* PCM 2822, with an IC₅₀ value of 0.02 µM
- Circular dichroism (CD) analysis revealed no significant DNA interaction by compound 8a, which is crucial and suggests its potential for in vivo applications without negative impact on host cells
- Molecular modeling indicated that the S isomer of compound 8a is preferred, and compound 8a binds best to Ser578 in the SufA active site, stabilized by intermolecular hydrogen bonds, salt bridge type bonds, and π-π interactions
- Compounds 8b and 8c showed lower SufA inhibitory activity than compound 8a
- Compound 8c displayed the lowest inhibitory potency toward SufA protease among the tested inhibitors, despite the strong electron-withdrawing properties of its substituent. Nevertheless, compound 8c showed a strong antibacterial effect against *S. marcescens* PCM 549 (IC₅₀ < 0.01 µM), which is more effective than commercially used gentamicin (IC₅₀ = 0.2506 µM)
- Compound 8c also demonstrated a strong antibacterial effect against *E. coli* PCM 2561
- Substitution at the phenyl diester ring did not drastically enhance activity toward SufA
- SufA inhibitors, such as compound 8a, are promising antibacterial agents, offering a potential avenue to combat antibiotic resistance
- Future research will focus on designing and synthesizing peptide-based derivatives incorporating the phosphonate warhead to further improve inhibitory potency and enhance biological efficacy, as well as optimizing physicochemical properties for potential in vivo applications

## Writing assistance

No writing assistance was utilized in the production of this manuscript.
