# Supplementary material for: Novel strategies for inhibiting SufA protease: the role of ester substituents and biological properties
Source: Future Med Chem. 2025 Aug 20;17(15):1827–37. doi: 10.1080/17568919.2025.2545173 (PMC12380226; doi:10.1080/17568919.2025.2545173)
Supplement: Supplemental Material [file IFMC_A_2545173_SM5605.docx]

Supporting information

Novel Strategies for Inhibiting SufA Protease: the Role of Ester Substituents and Biological Properties

Ewa Burchacka*, Paweł Pięta, Katarzyna Pstrowska, Agnieszka Korzenowska-Kowal, Gabriela Cieniuch, Michał Jewgiński

Ewa Burchacka: Department of Organic and Medicinal Chemistry, Faculty of Chemistry, Wrocław University of Science and Technology, 27 Wybrzeże Wyspiańskiego St, 50-370 Wrocław, Poland, E-mail: ewa.burchacka@pwr.edu.pl

Paweł Pięta: Department of Cell Biology, Poznan University of Medical Sciences, 10 A. Fredry St, 61-701 Poznań, Poland

Katarzyna Pstrowska: Department of Advanced Material Technologies, Faculty of Chemistry, Wrocław University of Science and Technology, Gdańska 7/9, 50-344 Wrocław, Poland

Agnieszka Korzenowska-Kowal, Gabriela Cieniuch:Polish Collection of Microorganisms, Department of Immunology of Infectious Diseases, Hirszfeld Institute of Immunology and Experimental Therapy, Polish Academy of Sciences, St. Weigla 12, 53-114 Wrocław, Poland

Michał Jewgiński: Department of Bioorganic Chemistry, Faculty of Chemistry, University

of Science and Technology, 27 Wybrzeże Wyspiańskiego St, 50-370 Wrocław, Poland

Table of Contents

**Chemical Part**

S1 Synthesis procedure for 6-formyl-2-naphthonitrile (**6**)

S2 Synthesis of Cbz-6-AmNphth^P^(OC_6_H_4_-4-R_1_)_2_ (**8a-c**)

**Microbiology Part**

S1 Antibacterial test of gentamycine toward *F. magna*, *S. aureus, E. coli* and *S. marcescens*

**Biochemical Part**

S1 Raw gel of results of inhibition of SufA-induced degradation of human fibrinogen by tested inhibitors after SDS PAGE electophoresis analysis

S2 Raw gel of results of inhibition of SufA-induced degradation of human LL-37 by tested inhibitor **8a** after SDS PAGE electophoresis analysis

**Molecular Modeling Part**

S1 Formation of phosphonate ester from condensation of ligand phosphonate with receptor alcohol

**Supplemental Figures**

Figure S1 HPLC purity analysis and ^1^H NMR for the 6-(Methoxycarbonyl)-2-naphthoic acid (**2**)

Figure S2 HPLC purity analysis and ^1^H NMR for the Methyl 6-carbamoyl-2-naphthoate (**3**)

Figure S3 HPLC purity analysis and ^1^H NMR for the Methyl 6-cyano-2-naphthoate (**4**)

Figure S4 HPLC purity analysis and ^1^H NMR for the 6-(Hydroxymethyl)-2-naphthonitrile (**5**)

Figure S5 ^1^H, ^31^P NMR, MS and HPLC purity analysis for the Cbz-6-AmNphth^P^(OC_6_H_4_-4-SCH_3_)_2_ (**8a**)

Figure S6 ^1^H, ^31^P NMR, MS and HPLC purity analysis for the Cbz-6-AmNphth^P^(OC_6_H_4_-4-OCH_3_)_2_ (**8b**)

Figure S7 ^1^H, ^31^P NMR, MS and HPLC purity analysis for the Cbz-6-AmNphth^P^(OC_6_H_4_-4-COOCH_3_)_2_ (**8c**)

Figure S8 General synthesis procedure for compunds Cbz-6-AmNphth^P^(OC_6_H_4_-4-R_1_)_2_ (**8a-c**)

Figure S9 Inhibition of SufA-induced degradation of human fibrinogen by tested inhibitors

Figure S10 Inhibition of SufA-induced degradation of LL-37 by tested inhibitor **8a**

Figure S11 Confirmation of the band coming from inhibitor **8a**

Figure S12 Effect of the gentamycine on the bacterial grow reduction with IC_50_ values

**Supplemental Tables**

Table S1 Full results of covalent docking of the investigated inhibitors including detailed structure od docked ligands.

**Chemical Part**

S1 Synthesis procedure for 6-formyl-2-naphthonitrile

**6-(Methoxycarbonyl)-2-naphthoic acid** (**2**) The dimethyl naphthalene-2,6-dicarboxylate (0.02 mol) was heated at 80-90°C in 1,4-dioxane (30 mL) until completely dissolved. Next, the solution of KOH in MeOH (1.32g KOH/10 mL MeOH) was added dropwise and the mixture was heated for 2h at 90°C. Then the crystalline product was filtrated, washed with diethyl ether, dried and dissolved in water (250 mL). The insoluble material was removed by filtration and the product was participated with 2M HCl, filtered, washed and dried under vacuum over P_2_O_5_. HPLC purity analysis and ^1^H NMR for the 6-(Methoxycarbonyl)-2-naphthoic acid (**2**) were presented on Figure S1.


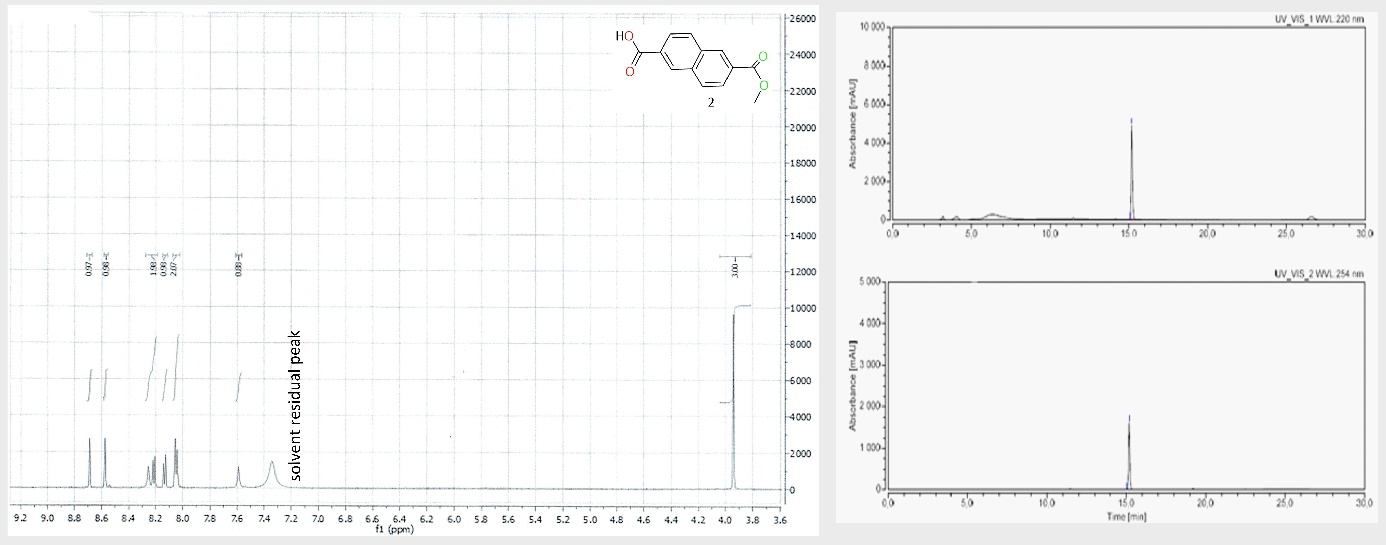


Figure S1 ^1^H NMR (left) and HPLC purity analysis (right) for the 6-(Methoxycarbonyl)-2-naphthoic acid (**2**)

**Methyl 6-carbamoyl-2-naphthoate** (**3**)The solution of 6-(methoxycarbonyl)-2-naphthoic acid (0.016 mol) and thionyl chloride (60 ml) in ethylene chloride (120 ml) was heated at 75°C for 3h. The volatile components were removed under reduced pressure, redissolved in dry toluene and evaporated again. Next, the oily residue was dissolved in anhydrous methylene chloride (40 ml) and 7M ammonia solution in methanol (5 ml) was added. The reaction was performed for 1h at room temperature. The product which precipitated as a white solid was filtrated and dried over P_2_O_5_.


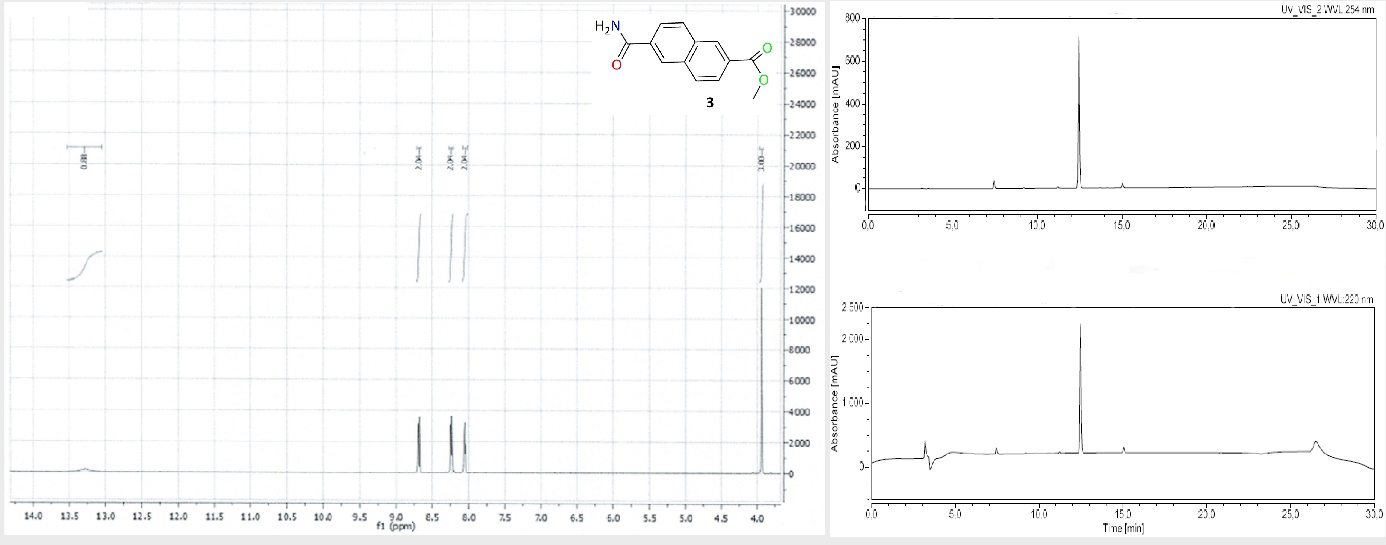


Figure S2 ^1^H NMR (left) and HPLC purity analysis (right) for the Methyl 6-carbamoyl-2-naphthoate (**3**)

**Methyl 6-cyano-2-naphthoate** (**4**) Methyl 6-carbamoyl-2-naphthoate (0.01 mol) was suspended in 1,4-dioxane (35 ml).The solution was cooled to 0°C in the ice bath. Then pyridine (2.4 ml) and trifluoroacetic anhydride (20 ml) was added dropwise. The reaction mixture was allowed to worm to room temperature and was continued for 48 h. The reaction mixture was poured on water (300 ml) and was extracted with ethyl acetate (3×70 ml). Combined organic extracts were washed with water (4×100 ml) and dried over MgSO_4_. After filtration the volatile components were removed in vacuum and the resulting oil was dissolved in chloroform, passed through a pad of Silica Gel and evaporated to dryness yielding final product as white solid.


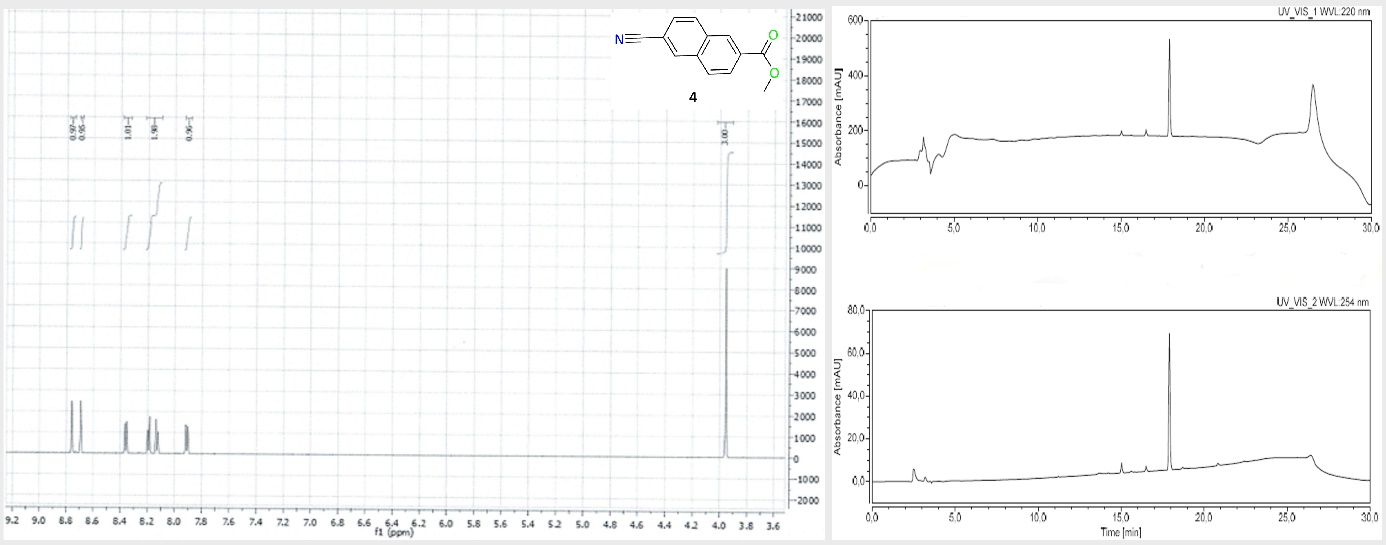


Figure S3 ^1^H NMR (left) and HPLC purity analysis (right) for the Methyl 6-cyano-2-naphthoate (**4**)

**6-(Hydroxymethyl)-2-naphthonitrile** (**5**) The methyl 6-cyano-2-naphthoate (0.008 mol) was suspend in THF (100 ml), The, the LiBH_4_ (0.09 mol) was added. The mixture was stirred for 30 min. at room temperature followed by the addition of ethyl alcohol (100 ml). The reaction was performed at room temperature for 24 h. Next, 5% aqueous citric acid solution was added dropwise and volatile components were removed under reduced pressure. The aqueous phase was extracted with chloroform (3×100 ml) and combined organic fractions were washed with water (2×100 ml), brine (2×100 ml) and dried over MgSO_4_. The solvent was evaporated under reduced pressure yielding desired white solid as 6-(Hydroxymethyl)-2-naphthonitrile.


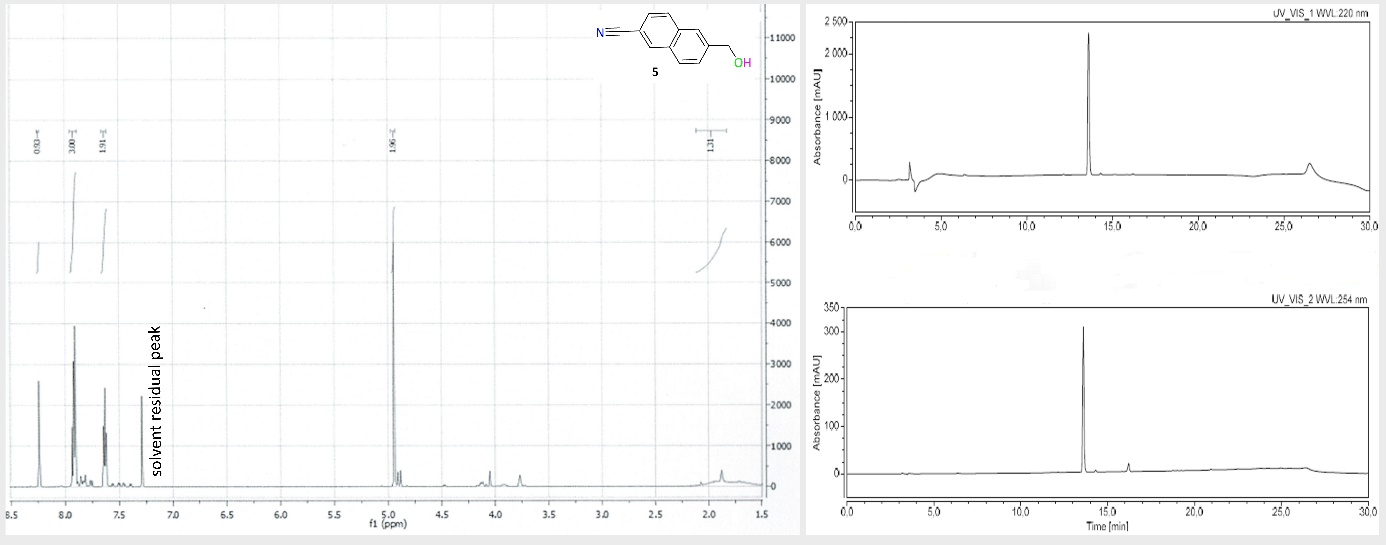


Figure S4 ^1^H NMR (left) and HPLC purity analysis (right) for the 6-(Hydroxymethyl)-2-naphthonitrile (**5**)

**6-Formyl-2-naphthonitrile** (**6**) Oxalyl chloride (0.1 mol) was dissolved in freshly dried dichloromethane (40 ml) and the solution was cooled to -60°C followed by a dropwise addition of dimethyl sulfoxide (0.27 mol) dissolved in 40 mL of dry dichloromethane. After 15 minutes 6-(Hydroxymethyl)-2-naphthonitrile (0.06 mol) dissolved in 100 mL of dry dichloromethane was added dropwise. The reaction was performed at -60°C for 2h. The reaction was quenched with triethylamine (2 mL) followed by the addition of saturated ammonium chloride solution (80 mL). The mixture was extracted with methylene chloride and combined organic fractions were washed with brine (50 mL) and dried over MgSO_4_. The solution was filtered and evaporated to dryness yielding crude aldehyde which was used directly in the amidoalkylation reaction.

S2 Synthesis of Cbz-6-AmNphth^P^(OC_6_H_4_-4-R_1_)_2_

**Cbz-6-CN-Nphth^P^(OC_6_H_4_-4-SCH_3_)_2_** (**7a**) The 6-formyl-2-naphthonitrile (0.011 mol), benzyl carbamate (0.011 mol) and P-(OC_6_H_4_-4-SCH_3_)_3_ (0.01 mol) were dissolved in 50 mL of glacial acetic acid. The reaction mixture was stirred at 80-90°C for 3 h. Volatile components were removed in vacuum. The crude product was crystallized from methanol at -20°C and was used in the next step without further purification.

**Cbz-6-AmNphth^P^(OC_6_H_4_-4-SCH_3_)_2_** (**8a**) Cbz-6-CN-Nphth^P^(OC_6_H_4_-4-SCH_3_)_2_ (1 mmol) was dissolved in a mixture of anhydrous ethyl alcohol (1.6 ml) and freshly dried chloroform (15 ml). Next, the solution was saturated with gaseous HCl and reaction was performed at -20°C for 4 days. Then product was precipitated with diethyl ether, filtered and dried in vacuum over P_2_O_5_. The obtained imino ether intermediate was dissolved in dry methanol (50 ml) and 7N ammonia solution in methanol (0.15 ml) was added. The reaction was performed at room temperature for 1 h. Subsequently, the volatile components of the reaction mixture were removed in vacuum. The resulting oil was redissolved in fresh methanol and refluxed for 8 h. The reaction mixture was evaporated and the oily product was further purified on SilicaGel using CHCl_3_:MeOH:AcOH/90:10:1, v/v/v) as the eluent yielding final product as a white solid.


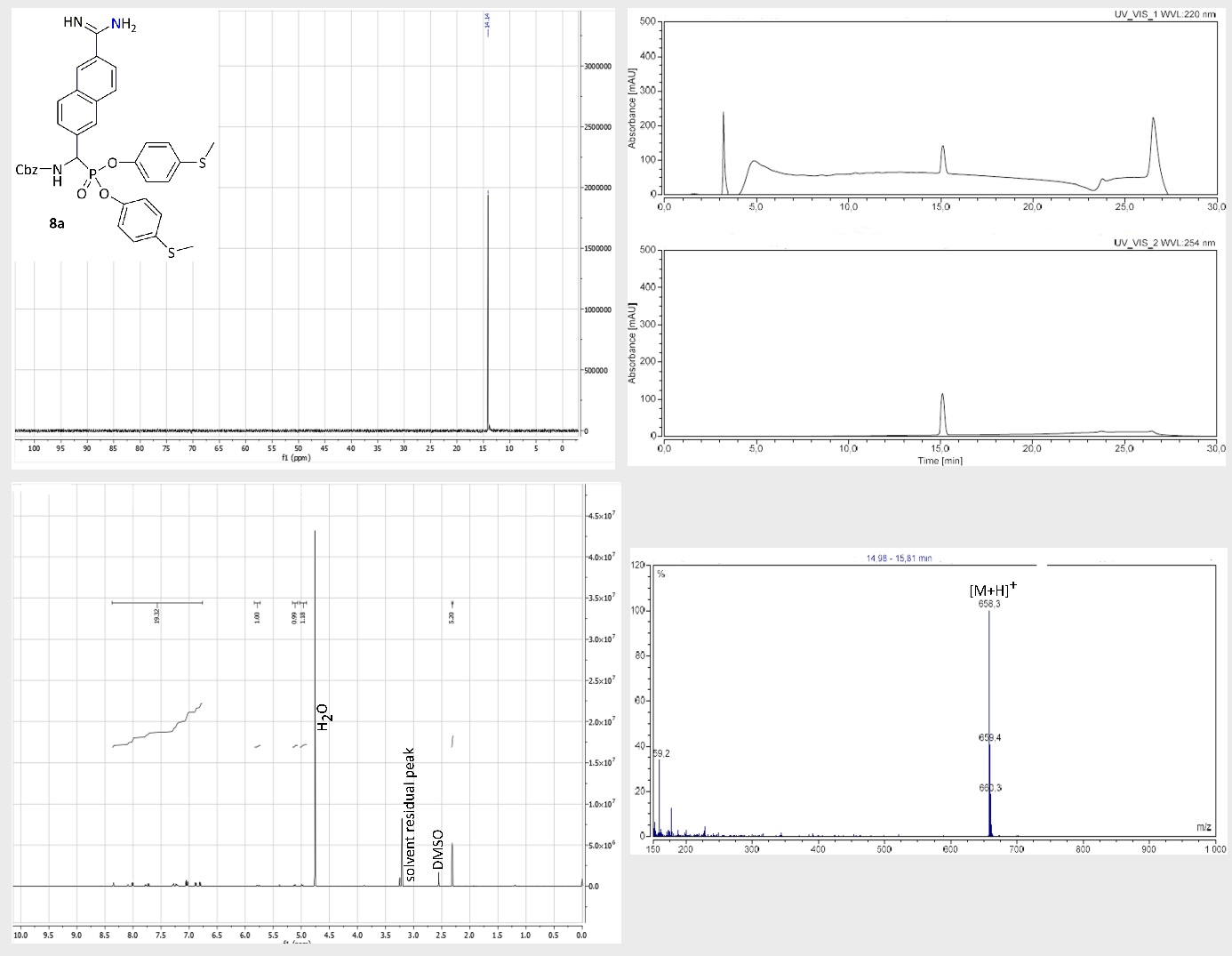


Figure S5 ^1^H (left bottom), ^31^P (left top) NMR, MS (right bottom) and HPLC purity (right top) analysis for the Cbz-6-AmNphth^P^(OC_6_H_4_-4-SCH_3_)_2_ (**8a**)

**Cbz-6-CN-Nphth^P^(OC_6_H_4_-4-OCH_3_)_2_** (**7b**) The 6-formyl-2-naphthonitrile (0.011 mol), benzyl carbamate (0.011 mol) and P-(OC_6_H_4_-4-OCH_3_)3 (0.01 mol) were dissolved in 50 mL of glacial acetic acid. The reaction mixture was stirred at 80-90°C for 3 h. Volatile components were removed in vacuum. The crude product was crystallized from methanol at -20°C and was used in the next step without further purification.

**Cbz-6-AmNphth^P^(OC_6_H_4_-4-OCH_3_)_2_** (**8b**) Cbz-6-CN-Nphth^P^(OC_6_H_4_-4-OCH_3_)_2_ (1 mmol) was dissolved in a mixture of anhydrous ethyl alcohol (1.6 ml) and freshly dried chloroform (15 ml). Next, the solution was saturated with gaseous HCl and reaction was performed at -20°C for 4 days. Then product was precipitated with diethyl ether, filtered and dried in vacuum over P_2_O_5_. The obtained imino ether intermediate was dissolved in dry methanol (50 ml) and 7N ammonia solution in methanol (0.15 ml) was added. The reaction was performed at room temperature for 1 h. Subsequently, the volatile components of the reaction mixture were removed in vacuum. The resulting oil was redissolved in fresh methanol and refluxed for 8 h. The reaction mixture was evaporated and the oily product was further purified on SilicaGel using CHCl_3_:MeOH:AcOH/90:10:1, v/v/v) as the eluent yielding final product as a white solid.


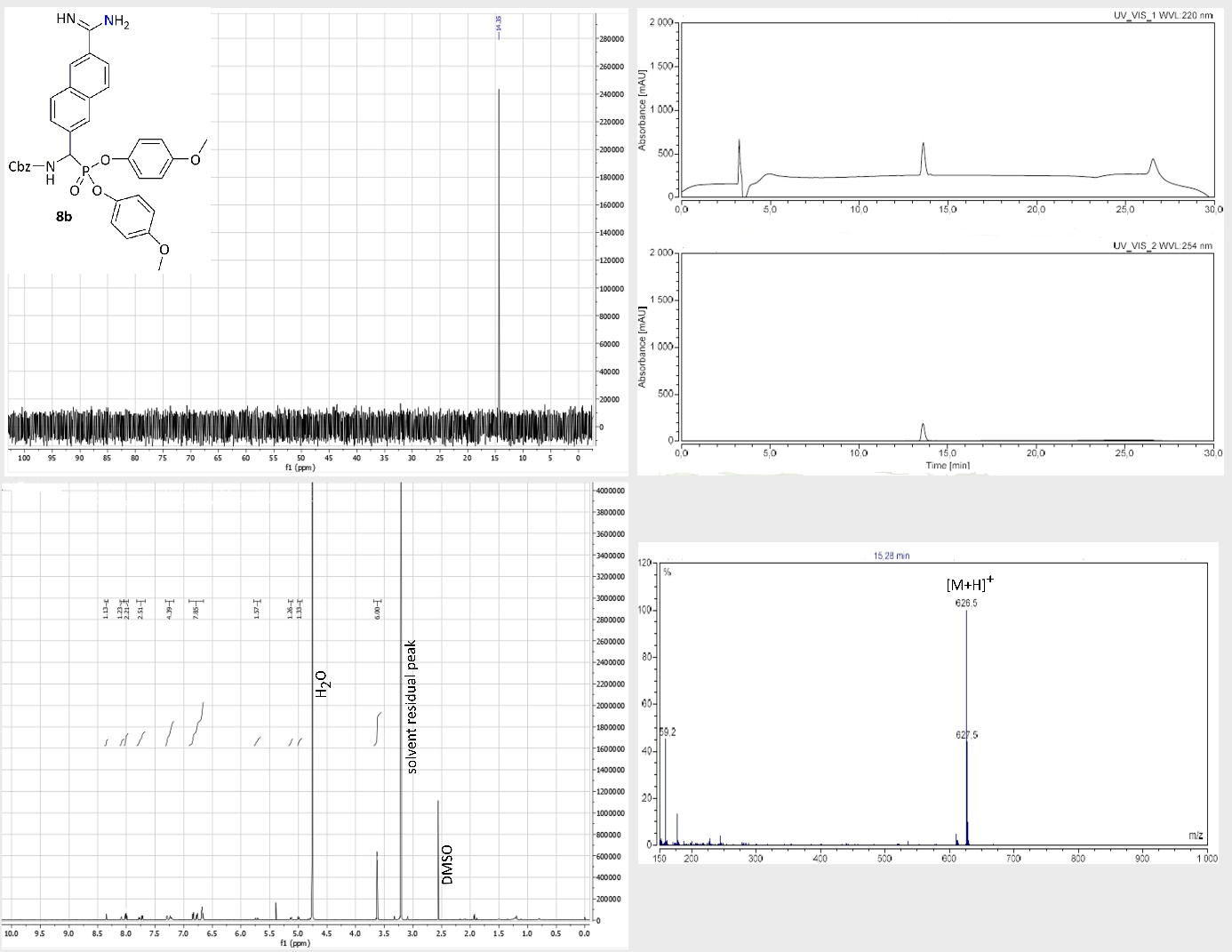


Figure S6 ^1^H (left bottom), ^31^P (left top) NMR, MS (right bottom) and HPLC purity (right top) analysis for the Cbz-6-AmNphth^P^(OC_6_H_4_-4-OCH_3_)_2_ (**8b**)

**Cbz-6-CN-Nphth^P^(OC_6_H_4_-4-COOCH_3_)_2_** (**7c**) The 6-formyl-2-naphthonitrile (0.011 mol), benzyl carbamate (0.011 mol) and P-(OC_6_H_4_-4-COOCH_3_)_3_ (0.01 mol) were dissolved in 50 mL of glacial acetic acid. The reaction mixture was stirred at 80-90°C for 3 h. Volatile components were removed in vacuum. The crude product was crystallized from methanol at -20°C and was used in the next step without further purification.

**Cbz-6-AmNphth^P^(OC_6_H_4_-4-COOCH_3_)_2_** (**8c**) Cbz-6-CN-Nphth^P^(OC_6_H_4_-4-COOCH_3_)_2_ (1 mmol) was dissolved in a mixture of anhydrous ethyl alcohol (1.6 ml) and freshly dried chloroform (15 ml). Next, the solution was saturated with gaseous HCl and reaction was performed at -20°C for 4 days. Then product was precipitated with diethyl ether, filtered and dried in vacuum over P_2_O_5_. The obtained imino ether intermediate was dissolved in dry methanol (50 ml) and 7N ammonia solution in methanol (0.15 ml) was added. The reaction was performed at room temperature for 1 h. Subsequently, the volatile components of the reaction mixture were removed in vacuum. The resulting oil was redissolved in fresh methanol and refluxed for 8 h. The reaction mixture was evaporated and the oily product was further purified on SilicaGel using CHCl_3_:MeOH:AcOH/90:10:1, v/v/v) as the eluent yielding final product as a white solid.


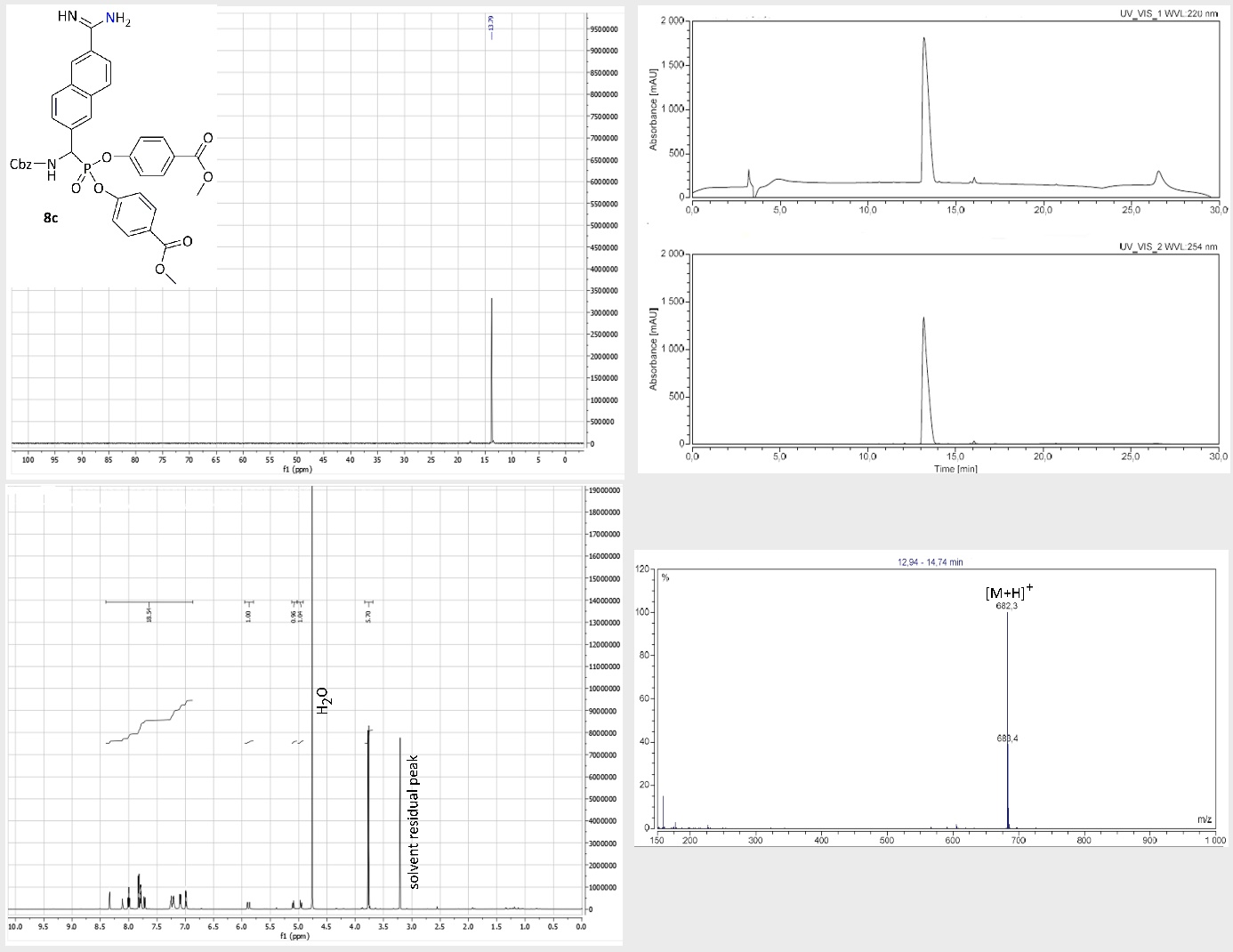


Figure S7 ^1^H (left bottom), ^31^P (left top) NMR, MS (right bottom) and HPLC purity (right top) analysis for the Cbz-6-AmNphth^P^(OC_6_H_4_-4-COOCH_3_)_2_ (**8c**)


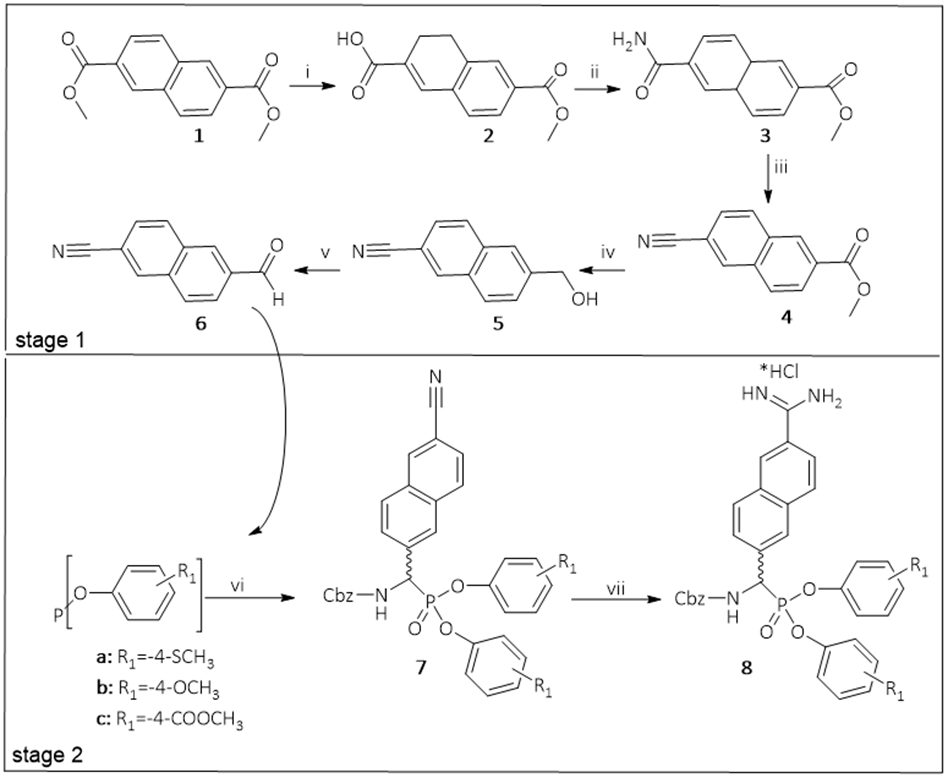


Figure S8 General synthesis procedure for compunds Cbz-6-AmNphthP(OC6H4-4-R1)2 (8a-c). Stage 1: synthesis of 6-formyl-2-naphthonitrile (6); i) KOH/MeOH, 1,4 dioxane, reflux ii) (CH2)2(Cl)2, SOCl3, reflux iii) 1,4-dioxane, pirydyne, TFAA, 0°C iv) LiBH4, THF, r.t v) oxalyl chloride, CH2Cl2, DMSO, Et3N, -70°C. Stage 2: synthesis of final inhibitors 8a-c; vi) benzyl carbamate, AcOH, reflux vii) HCl, CHCl3, EtOH, NH3/MeOH.

**Biochemical Part**

S1 Raw gel of results of inhibition of SufA-induced degradation of human fibrinogen by tested inhibitors after SDS PAGE electophoresis analysis


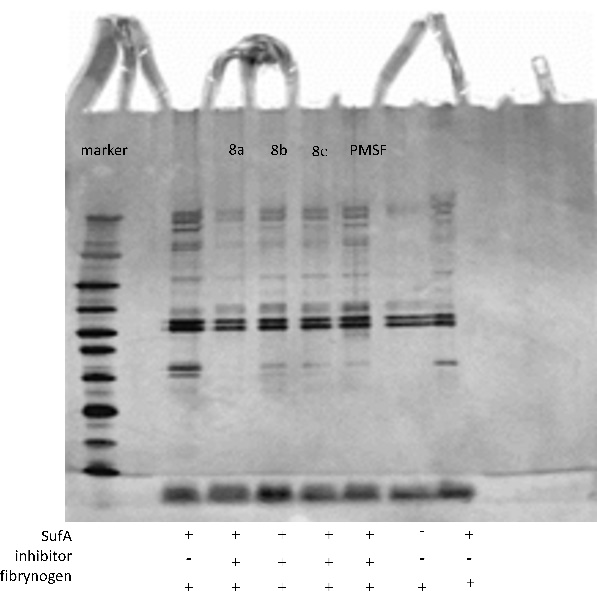


Figure S9 Inhibition of SufA-induced degradation of human fibrinogen by tested inhibitors

S2 Raw gel of results of inhibition of SufA-induced degradation of human LL-37 by tested inhibitor **8a** after SDS PAGE electophoresis analysis


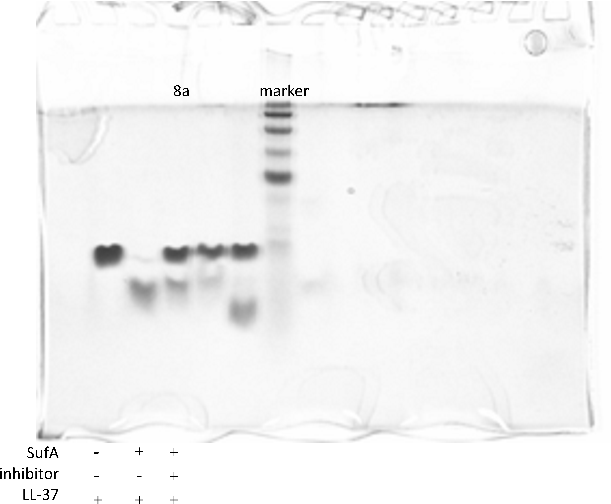


Figure S10 Inhibition of SufA-induced degradation of LL-37 by tested inhibitor **8a**


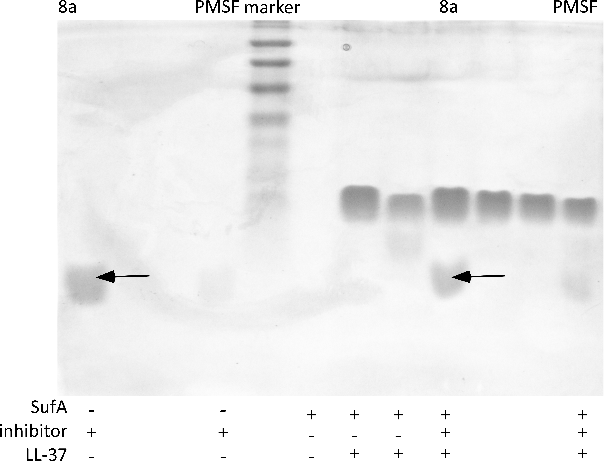


Figure 11 Confirmation of the band coming from inhibitor **8a**

**Microbiology Part**

S1 Antibacterial test of gentamycine toward *F. magna*, *S. aureus*, *E. coli* and *S. marcescens*

Figure S12 Effect of the gentamycine on the bacterial grow reduction with IC_50_ values

**Molecular Modeling Part**

S1 Formation of phosphonate ester from condensation of ligand phosphonate with receptor alcohol

# Receptor CYS/SER, sulfur/oxygen is <1>

RECEPTOR_SMARTS_PATTERN 2,[C,c]-[S,O;H1,-1]

# Ligand, phosphorus is <2>

LIGAND_SMARTS_PATTERN 1,[P]-[O;H1,-1]

# Neutralize the CYS/SER, if necessary

CUSTOM_CHEMISTRY ("<1>",("charge",0,(1)))

# Delete the ligand hydroxyl

CUSTOM_CHEMISTRY ("<2>-[O;H1,-1]",("delete",2))

# Add ligand-receptor single bond

CUSTOM_CHEMISTRY ("<1>|<2>",("bond",1,(1,2)))

Table S1 Full results of covalent docking of the investigated inhibitors including detailed structure od docked ligands.

| Name | Structure | Score glide e-model | Docking score | MMGBSA dG Bind* | MMGBSA dG Bind* - average of all isomers | MMGBSA dG Bind (NS)** | MMGBSA dG Bind (NS)** - average of all isomers | H-bond | Salt Bridges |
| --- | --- | --- | --- | --- | --- | --- | --- | --- | --- |
| **8a**_R1 |  | -81.247 | -6.697 | -73.35 | -81,99 | -96.37 | -99,93 | 1 | 2 |
| **8a**_R2 |  | -90.015 | -5.538 | -73.73 |  | -84.56 |  | 1 | 0 |
| **8a**_S1 |  | -90.131 | -6.753 | -92.33 |  | -111.96 |  | 7 | 2 |
| **8a**_S2 |  | -79.784 | -6.733 | -88.53 |  | -106.82 |  | 3 | 2 |
| **8b**_R1 |  | -73.259 | -5.285 | -60.11 | -76,80 | -76.21 | -93,46 | 1 | 0 |
| **8b**_R2 |  | 12.628 | -5.395 | -75.81 |  | -91.32 |  | 2 | 2 |
| **8b**_S1 |  | -92.164 | -8.526 | -89.38 |  | -106.92 |  | 7 | 2 |
| **8b**_S2 |  | -91.146 | -6.929 | -81.91 |  | -99.40 |  | 3 | 2 |
| **8c**_R1 |  | 0.000 | 8.695 | -71.21 | -74,35 | -86.57 | -91,46 | 3 | 0 |
| **8c**_R2 |  | -99.668 | -5.925 | -68.05 |  | -79.70 |  | 2 | 0 |
| **8c**_S1 |  | -85.028 | -7.097 | -84.45 |  | -105.24 |  | 4 | 2 |
| **8c**_S2 |  | -63.040 | -5.765 | -76.85 |  | -94.32 |  | 3 | 2 |

* ) MMGBSA dG Bind = Complex Receptor – Ligand

**) MMGBSA dG Bind(NS) = Complex Receptor (from the optimized complex) – Ligand (from optimized complex)
